# A valid model for predicting responsible nerve roots in lumbar degenerative disease with diagnostic doubt

**DOI:** 10.1186/s12891-016-0973-3

**Published:** 2016-03-15

**Authors:** Xiaochuan Li, Xuedong Bai, Yaohong Wu, Dike Ruan

**Affiliations:** Department of Orthopedic, Navy General Hospital, NO. 6 Fucheng Road, Beijing, 100048 China; Department of Orthopedic, Gaozhou people’s Hospital, Guangdong, China

**Keywords:** Lumbar degenerative disease, Diagnostic doubt, Predictive model, Selective nerve route block

## Abstract

**Objective:**

To construct and validate a model to predict responsible nerve roots in lumbar degenerative disease with diagnostic doubt (DD).

**Methods:**

From January 2009-January 2013, 163 patients with DD were assigned to the construction (*n* = 106) or validation sample (*n* = 57) according to different admission times to hospital. Outcome was assessed according to the Japanese Orthopedic Association (JOA) recovery rate as excellent, good, fair, and poor. The first two results were considered as effective clinical outcome (ECO). Baseline patient and clinical characteristics were considered as secondary variables. A multivariate logistic regression model was used to construct a model with the ECO as a dependent variable and other factors as explanatory variables. The odds ratios (ORs) of each risk factor were adjusted and transformed into a scoring system. Area under the curve (AUC) was calculated and validated in both internal and external samples. Moreover, calibration plot and predictive ability of this scoring system were also tested for further validation.

**Results:**

Patients with DD with ECOs in both construction and validation models were around 76 % (76.4 and 75.5 % respectively). Risk factors: more preoperative visual analog pain scale (VAS) score (OR = 1.56, *p* < 0.01), stenosis levels of L4/5 or L5/S1 (OR = 1.44, *p* = 0.04), stenosis locations with neuroforamen (OR = 1.95, *p* = 0.01), neurological deficit (OR = 1.62, *p* = 0.01), and more VAS improvement of selective nerve route block (SNRB) (OR = 3.42, *p* = 0.02). Validation: the internal area under the curve (AUC) was 0.85, and the external AUC was 0.72, with a good calibration plot of prediction accuracy. Besides, the predictive ability of ECOs was not different from the actual results (*p* = 0.532).

**Conclusions:**

We have constructed and validated a predictive model for confirming responsible nerve roots in patients with DD. The associated risk factors were preoperative VAS score, stenosis levels of L4/5 or L5/S1, stenosis locations with neuroforamen, neurological deficit, and VAS improvement of SNRB. A tool such as this is beneficial in the preoperative counseling of patients, shared surgical decision making, and ultimately improving safety in spine surgery.

## Introduction

Lumbar degenerative disease (LDD) often displayed as multilevel degeneration and stenosis occurs due to compression or ischemia, or both, of the lumbosacral nerve roots as a consequence of osteoarthritic thickening of the articulating facet joints, infolding of the ligamentum flava, and degenerative bulging of the intervertebral discs [1–3]. It is the main cause of chronic low back pain in old people, leading to spine surgery among individuals older than 65 years [4, 5]. With an increase in aging population, the number of people who suffer from this condition is expected to grow exponentially and this will have a significant effect on healthcare resources in the near future. Surgical decompression of the responsible compression sites remains as a widely accepted therapy of LDD currently [6–10].

Interestingly, although most patients with LDD exhibit a typical painful experience or present obvious degenerative changes on computed tomography or magnetic resonance imaging (MRI) scans, there still exists a group of patients with LDD whose diagnosis are uncertain or who have an ambiguous compressive region. In other words, when the responsible nerve roots are vague, or the pain source does not correspond to typical classical dermatomal patterns [11, 12], it is very difficult to select the decompression site and make reasonable surgical plans for such patients with diagnosis doubt (DD). Moreover, to date, there are no studies proposing a predictive method to determine responsible nerve roots in patients with DD. Nonetheless, studies on this topic are still in progress. Recently, an increasing amount of evidence has demonstrated selective nerve root block (SNRB) may play a role in predicting the responsible compression nerve roots [13–18]. However, LDD usually reported a complicated progress, involving multiple factors, such as stenosis levels [19], stenosis locations [20], neurological deficit [21], and preoperative Oswestry disability index (ODI) score [22], that makes it quite complex to distinguish the responsible nerve roots.

Therefore, in this case, we planned to use relevant parameter of SNRB combined with some risk factors screened out from the baseline patient-related factors and clinical characteristics to establish a scoring system through multivariable logistic model. After that, the utility of this new predictive model was examined in an external subpopulation of a validation sample. Ultimately, we hope this new predictive model will play a role in decision making of which segments should to be decompressed and how many decompression segments should be conducted in such patients with DD.

## Materials and methods

### Research institution

The study was undertaken in the Department of Orthopedics of two hospitals (Navy General Hospital, Beijing, China, and Gaozhou people’s Hospital, Guangdong, China).

### Study design

We conducted a study evaluating whether baseline patient and clinical characteristics could distinguish responsible nerve roots of LDD patients with DD. Briefly, primary outcome measures included visual analog pain scale (VAS) score (0–10 points), ODI, Japanese orthopedic association (JOA) score (0–29 points), the diagnostic test of SNRB, and imaging information.

### The inclusion and exclusion criteria

Patients with DD were retrospectively and consecutively reviewed from January 2009 –January 2013. The inclusion criteria were as follows: (a) Patients diagnosed as LDD. (b) The physical examinations, radiography, MRI scans, and SNRB were all conducted for a definite diagnosis. (c) All tests of VAS score (0–10 points), ODI and JOA score (0–29 points) were evaluated and available. (d) The main characteristics of these patients were that the responsible nerve roots or pain source were difficult to be distinguished, or physical examination did not correspond to imaging scan. (e) Patients had undergone laminectomy decompression and were followed clinically for a minimum period of 24 months. The exclusion criteria included lumbar spinal stenosis caused by spondylolisthesis, tumor, deformity, osteoporosis and infection.

### Statistical methods

The quantitative variables were described by mean and standard deviations and the qualitative variables by absolute and relative frequencies. All the analyses were performed at a significance level of 5 % and the associated confidence intervals (CIs) were estimated for each relevant parameter. All the analyses performed by using IBM SPSS Statistics 19.0. Mann–Whitney U test or Pearson *χ*2 test (according to the type of variable) were used to verify differences in patient baseline and clinical characteristics.

In the construction sample, a multivariate logistic regression model was made with outcome as the dependent variable and the other study variables as explanatory variables. The receiver operating characteristic (ROC) curve was calculated and the following points determined: [23, 24] (1) optimum: that which minimized the√([1-sensitivity]^2^þ[1-specificity]^2^); (2) discard: that which had a negative likelihood ratio (NLR) < 0.1, or the left-tail probability < 5 % (value usually taken as a small error in medical statistics); and (3) confirmation: that with positive likelihood ratio (PLR) > 10 or, if this did not exist, that with right-tail probability > 55 % (value slightly greater than chance, 50 %). For each of the points calculations were made of the sensitivity, specificity, PLR and NLR. The following risk groups were defined: very low (<discard point), low (≥discard point and < optimum point), medium (≥optimum point and < confirmation point) and high (≥confirmation point).

### Ethical approval

The application for approval of human research protocol has been reviewed and approved by the Navy General Hospital Ethical Committee (NGHEC) NGHEC Approval No. 2015–0107.

## Results

To evaluate the responses of last JOA score after a minimum of 2 years follow-up, questionnaires were prepared to determine the percentage of patients with ECO or non-effective clinical outcome (NECO) relative to their initial questionnaire values. The clinical outcomes were divided into the following four types according to different JOA recovery rate which was calculated by the Hirabayashi method [25]: (postoperative score − preoperative score)/(29 − preoperative score) × 100 %. The four types of recovery rates were graded as follows: >75 %, excellent; 50–74 %, good; 25–49 %, fair; and <25 %, poor. The first two results were considered as ECOs.

Totally, of the 191 patients included in the study, 163 cases finally fulfilled the inclusion criteria, representing a loss of 14.7 % (*n* = 28), of whom, had at least one of the exclusion criteria. The 163 patients with DD were assigned to the construction sample (*n* = 106) or validation sample (*n* = 57) according to different admission times to hospital.

The baseline patient-related factors between the ECOs (excellent or good) and NECOs (fail or poor) on follow-up for no less than 2 years are compared in Table 1. The results showed no significant difference between the two groups (*P* = 0.08–0.87) (Table 1).

**Table 1:** Patient baseline demographic characteristics, comorbidities, and health status measures according to clinical outcome

	Clinical outcomes after 2 years		
	ECOs (*n* = 81)	NECOs (*n* = 25)	*P*
Mean age (SD)^c^	62.8 ± 9.5	59.4 ± 8.2	0.08
Female^b^	39	11	0.72
Ethnicity (Han)^a^	76	24	0.67
Education (at least some school)^a^	73	22	0.72
Marital status (married)^a^	77	23	0.63
Compensation (Any)^a^	74	21	0.29
Mean BMI (SD)^c^	20.7 ± 3.9	22.3 ± 3.7	0.11
Smoker (no)	27	8	0.81
Work status: no^a^			0.39
Full or part time	13	7	
Retired	27	6	
Other	41	12	
Self-assessed health trend: no^a^			0.12
Staying about the same	47	9	
Getting worse	24	13	
Other	10	3	
Comorbidities: no^a^			0.87
Hypertension	34	8	
Diabetes	12	4	
Osteoporosis	27	5	
Heart problem	19	4	
Stomach problem	15	6	
Bowel or intestinal problem	11	5	
Depression	7	3	
Joint problem	44	10	
Other	17	5	
Total number of comorbidities^a^			0.36
None	17	6	
One	29	4	
Two	22	9	
More than two	20	5	

*NECO* non-effective clinical outcome, *BMI* indicates body mass index, *SD* indicates standard deviation

^a^Fisher exact test; ^b^Pearson *χ* ^2^ test; ^c^Mann Whitney U test; ECO: effective clinical outcome

Additionally, a comparison of clinical characteristics between the ECOs and NECOs is presented in Table 2. The significant risk factors were stenosis level (L4/5, *P* = 0.02; L5/S1, *P* = 0.03), stenosis locations (neuroforamen, *P* = 0.03), neurological deficit (Reflexes, *P* = 0.03; Sensory, *P* = 0.02; Motor, *P* = 0.02), higher VAS score before operation (*P* = 0.01), and more VAS improvement rate after SNRB (*P* = 0.01). Our results of risk factors were mainly tallying with the previous report except for the higher VAS score before operation. This is a newly found risk factor in LDD patients with DD that may reflex the compression nerve roots to a certain extent.

**Table 2:** Patient Baseline of Clinical Characteristics

	Clinical outcomes after 2 years		
	ECOs (*n* = 81)	NECOs (*n* = 25)	*P*
Pseudoclaudication: any^b^	62	15	0.13
SLR or femoral tension^b^	20	8	0.51
Course of disease: yr^c^	3.2 ± 2.6	3.5 ± 3.1	0.64
Pain radiation: any^b^	57	18	0.88
Any neurological deficit			
Reflexes: asymmetric depressed^a^	32	4	0.03
Sensory: asymmetric decrease^a^	43	5	0.02
Motor: asymmetric weakness^a^	34	4	0.02
VAS improvement rate after SNRB^a^			0.01
≤24 %	8	7	
25 ~ 49 %	37	9	
50 ~ 74 %	21	3	
≥75 %	5	6	
ODI improvement rate after SNRB^a^			0.13
≤24 %	20	6	
25 ~ 49 %	45	9	
50 ~ 74 %	12	6	
≥75 %	4	4	
VAS score before operation^a^			0.01
≤2	12	9	
3 ~ 5	23	8	
5 ~ 7	27	4	
≥8	19	4	
ODI before operation^a^			0.32
≤24 %	25	6	
25 ~ 49 %	33	7	
50 ~ 74 %	18	9	
≥75 %	5	3	
X-ray of lumbar vertebra			
Degenerative scoliosisb^b^	25	7	0.79
Lumbar lordosis disappear^b^	72	19	0.19
Degenerative lumbar instability^b^	28	6	0.32
Stenosis level			
L1-L2^a^	10	4	0.74
L2-L3^a^	17	4	0.78
L3-L4^b^	59	15	0.22
L4-L5^a^	78	17	0.02
L5-S1^b^	32	4	0.03
Total number of stenosis^a^			0.72
Two	13	5	
Three	36	9	
More than 3	32	11	
Stenosis locations			
Central or Lateral recess^b^	71	18	0.11
Neuroforamen	21	5	0.03
Stenosis severity^b^			0.55
Mild	25	8	
Moderate	35	13	
Severe	21	4	

*NECO* non-effective clinical outcome, *VAS* Visual analog scale, *ODI* Oswestry Disability Index, *SLR* single leg raise

^a^Fisher exact test; ^b^Pearson *χ* ^2^ test; ^c^Mann Whitney U test; ECO: effective clinical outcome

After all risk factors were screened out, a multivariate logistic regression model was used with clinical outcome as the dependent variable, and the 5 risk factors as explanatory variables in the construction sample (Table 3). Thus, the respective odds ratio (OR) of risk factors were higher VAS score before operation (OR = 1.56, 95 % CI: 1.08–2.65, *P* < 0.01), stenosis levels of L4/5 or L5/S1 (OR = 1.44, 95 % CI: 1.10–1.89, *P* = 0.04), stenosis locations of neuroforamen (OR = 1.95, 95 % CI: 1.32–3.51, *P* = 0.01), neurological deficit (OR = 1.62, 95 % CI: 1.02–2.79, *P* = 0.01), and VAS improvement after SNRB (OR = 3.42, 95 % CI: 1.27–7.64, *P* = 0.02).

**Table 3:** Descriptive characteristics and analysis for determining responsible nerve roots of LDD patients with diagnostic doubt in both construction and validation samples

Variable	Construction sample	Validation sample	*P*-value	Adj. OR (95 % CI)	*P*-value
	(*n* = 106)	(*n* = 57)			
	*n* (%)/x ± s	*n* (%)/x ± s			
ECO	81	43	0.889	N/M	N/M
VAS score before operation^▲^			0.210	1.56 (1.08–2.65)	0.001
≤2	21	9			
3 ~ 5	31	12			
5 ~ 7	41	15			
≥8	23	21			
Stenosis level				1.44 (1.10–1.89)	0.04
L1-L2^▲^	14	5	0.400		
L2-L3^▲^	21	6	0.128		
L3-L4^▲^	64	27	0.111		
L4-L5^▲^	92	44	0.116		
L5-S1^▲^	36	12	0.085		
Stenosis locations				1.95 (1.32–3.51)	0.01
Central^▲^	73	36	0.460		
Lateral recess^▲^	82	49	0.130		
Neuroforamen^▲^	29	14	0.250		
Neurological deficit				1.62 (1.02–2.79)	0.01
Reflexes: asymmetric depressed^▲^	36	16	0.442		
Sensory: asymmetric decrease^▲^	48	19	0.139		
Motor: asymmetric weakness^▲^	38	23	0.571		
VAS score improvement after SNRB^▲^			0.762	3.42 (1.27–7.64)	0.02
≤24 %	15	9			
25 ~ 49 %	46	25			
50 ~ 74 %	24	13			
≥75 %	11	10			

^▲^ indicates significant difference, *LDD* lumbar degenerative disease, *SNRB* selective nerve root block, *N*/*M* not in the model, *Adj. OR* adjusted odds ratio, *CI* confidence interval, *VAS* Visual analog scale

Once the logistic regression model was constructed, this was transformed into a scoring system according to the OR of each risk factor (Table 4). The key points that defined the risk groups were as follows. 1) Discard: value, 5; sensitivity, 0.98 (95 % CI: 0.95–1.00); specificity, 0.09 (95 % CI: 0.05–0.14); PLR, 2.03 (95 % CI: 1.93–2.77); NLR, 0.43 (95 % CI: 0.24–0.65). 2) Optimum: value, 11; sensitivity, 0.86 (95 % CI: 0.82–0.93); specificity, 0.67 (95 % CI: 0.56–0.75); PLR, 2.46 (95 % CI: 2.13–2.85); NLR, 0.46 (95 % CI: 0.22–0.57). 3) Confirmation: value, 16; sensitivity, 0.13 (95 % CI: 0.06–0.21); specificity, 0.99 (95 % CI: 0.97–1.00); PLR, 3.39 (95 % CI: 2.64–3.93); NLR, 0.61 (95 % CI: 0.42–0.79).

**Table 4:** The model to predict responsible nerve roots in LDD patients with diagnostic doubt

A		B		C		D		E		Total score	Result	Risk (%)
Stenosis locations	Score	VAS score	Score	Neurological deficit	Score	VAS improvement	Score	SNRB nerve roots	Score			
Neuroforamen	4	≥8	3	Motor	1	≥75	7	L4/5 Or L5/S1	3	≥16	Very high	≥89.3
		5 ~ 7	2	Sensory	1	50 ~ 74	5			11 ~ 16	High	57.6 ~ 82.7
Lateral recess or Central	0	3 ~ 5	1	Reflexes	1	25 ~ 49	3	Others	0	5 ~ 10	Low	12.5 ~ 49.4
		≤2	0	None	0	≤24	0			<5	Very low	<8.6

*LDD* lumbar degenerative disease, *SNRB* selective nerve root block, *VAS* Visual analog scale

**Fig. 1:**
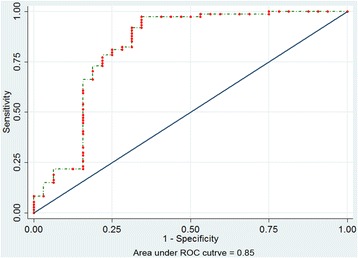
ROC curve of the model in construction sample (*n* = 106)

**Fig. 2:**
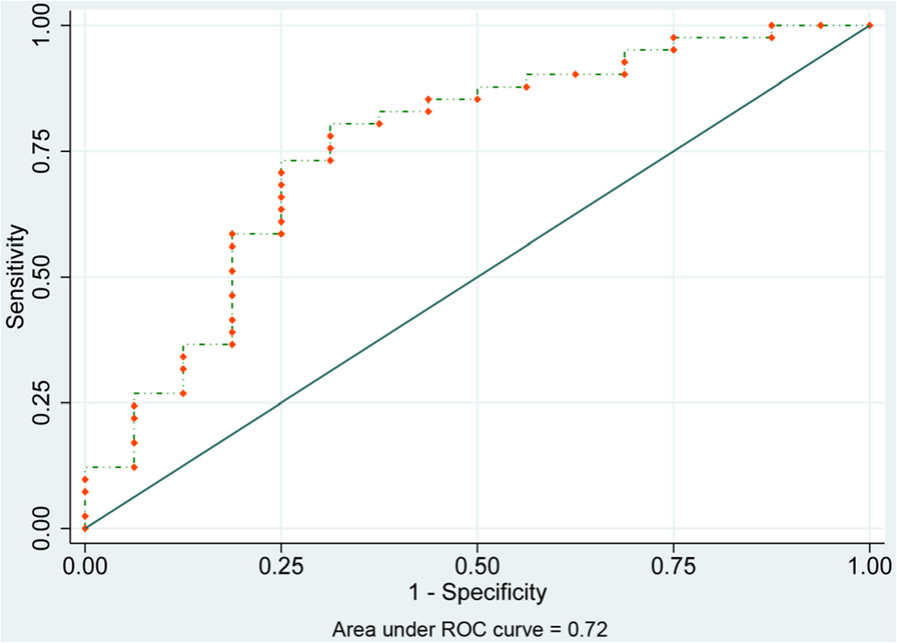
ROC curve of the model in validation sample (*n* = 57)

**Fig. 3:**
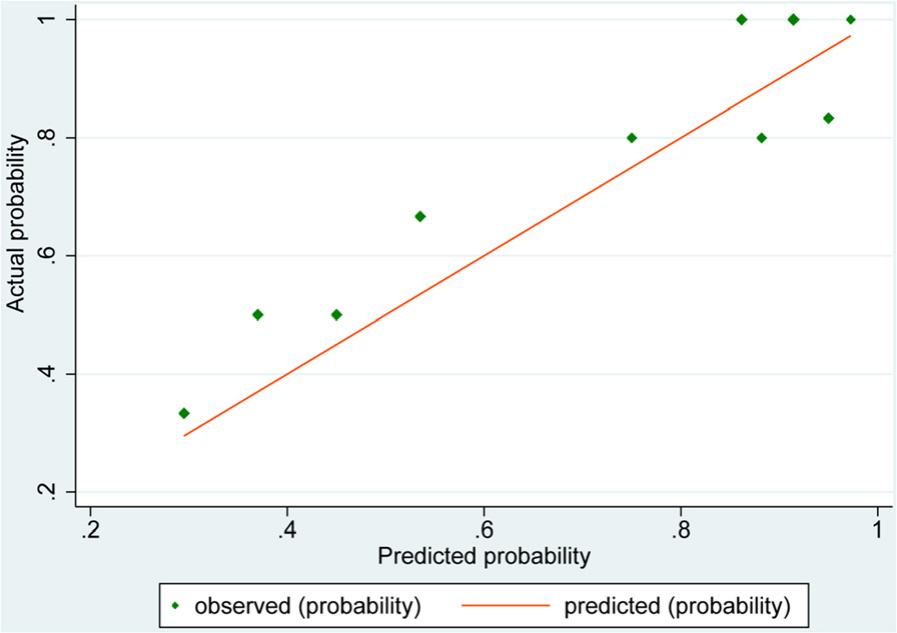
The calibration plot of the predictive model

**Fig. 4:**
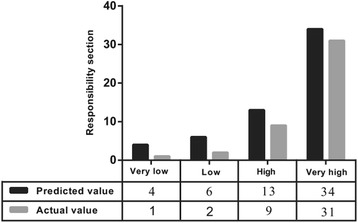
The comparison of predictive and actual outcomes of responsible nerve roots in LDD patients with DD

After the system score was established, we firstly tested it in the construction sample as an internal validation and the result of the area under curve (AUC) was 0.85, which demonstrated it to be a good model (Fig. 1). Moreover, the risk factors in the construction and validation samples were analyzed and the results were similar in both samples (*P* = 0.085–0.889) (Table 3). The ECOs of DD patients were around 76 % in the two groups (76.4 and 75.5 % respectively). On this basis, the ROC curve for our scoring system in the validation sample are reasonable and the AUC was 0.72 (Fig. 2). Additionally, to evaluate the calibration plot of this model, the data were also tested in the validation sample and the predicted probability showed good linear relationship with the actual probability, which exhibited as an appropriate calibration plot (Fig. 3). Finally, we once again compared the predicted and actually observed outcomes of this scoring system, and the analyzed result show no significant difference within the two samples (*P* = 0.532, Fig. 4).

## Discussion

This study constructed and validated a predictive model to determine the responsible nerve roots in DD patients. This model was constructed by transforming complex factors into a simple scoring system to enable a rapid calculation. To the best of our knowledge, this is the first predictive model in clinical application. In this model, both the internal and external AUC were > 0.7 and the calibration plot of prediction accuracy were tested as a good linear relationship. In addition, the predictive and actual outcomes showed no significant difference. Hence, this model was applicable and valid. As is known to all, LDD often displays several segmental pathological changes without exact localizing signs on physical examination, because of its elusive symptoms and missing standards on imaging analysis [26]. When counseling a patient with DD on which segments to be decompressed, or how many decompression segments to be conducted, a predictive model such as this, is of paramount importance.

In addition, a predictive model like this is also beneficial when considering risk factors. In our study, the univariate logistic regression model suggested 5 risk factors including higher VAS score before operation, stenosis levels of L4/5 or L5/S1, stenosis locations of neuroforamen, neurological deficit, and VAS improvement after SNRB. This will also play some role in some other kinds of lumbar spine diseases like failed back syndrome. Moreover, this model is a useful adjunct in predicting the clinical outcome after decompression surgery [13, 27, 28]. In our analysis, the OR of VAS improvement after SNRB was 3.42, which played a major role in the model. At this point, whether the pain relief after surgical decompression is good could be forecasted by this test to a certain extent because of the evidence that pain originating from nerve root compression can be effectively treated by surgical decompression [29–31]. Nevertheless, our model also combines together several other related risk factors in order to improve the predictive accuracy, because only SNRB is not a cost-effective method for identifying the symptomatic nerve roots [27].

Our model was built upon JOA recovery rate with a minimum following-up of 24 months. Although this cannot replace long-term follow-up results and ultimate outcomes, our conclusions are based on the curative effect, and this model is supported by comprehensive evidence of credible outcomes in clinical trials. Meanwhile, this model also cannot draw any definite conclusion. At least, when the score in our model is >16 points or <5 points, we may get a rational and objective reason about whether it should be considered as a responsible segment or not. Additionally, this model could be used as a reference index in patients with DD for arriving at a diagnosis and for treatment purposes.

Since this model was based on the SNRB test, we would like to recommend the following guidelines: 1). Surgeons should be familiar with the anatomy so that he or she could accurately determine the precise nerve root of the test; 2). It is still important to preliminarily identify the possible responsible segment by combination of detailed physical examination and radiological results before SNRB; 3) In a possible liability gap, the most likely responsible segment should be tested first rather than one by one. If symptoms were relieved by >50 %, it could be judged as the responsible gap, or else taking order from the lowest nerve roots, because the block of upper nerve root is prone to defuse to the lower one, and thus, interferes with the result. 4). Needle should be introduced gradually under fluoroscopic guidance to avoid unnecessary nerve root injury and 5). The single dose should not be too much, generally 1 % lidocaine 0.5–1 ml, otherwise it will also cause other nerve roots.

As with any study, there are limitations to the present study. First, a great number of variations exist and we possibly did not identify all significant variables to predict the result. Future studies of this model may consider the effect of a more detailed database that contains more input variables (such as electromyography and the walking distance). Second, the number of patients was relatively small and this may have prevented significant correlation between the two groups. Finally, many subjective grading scores were not performed by the same surgeon on the same patient, and that may introduce some errors between the groups. However, we did attempt to minimize the weaknesses by using strict criteria for inclusion and exclusion. Although we were also very strict while performing the case inclusion criteria, these differences might be reduced but not abolished. Nevertheless, our model was validated, so that precise predictions are possible.

## Conclusions

In summary, this study constructed and validated a predictive model that can be used to determine responsible segments or pain source of patients with DD. This tool is of substantial value in the preoperative counseling of patients, shared surgical decision making, and ultimately improving safety in spine surgery. Second, as we progress into an era of quality metrics and performance assessment, a tool like this can be beneficial in risk adjustment. Future predictive models are recommended for further risk stratification and modification.

## Abbreviations

AUC: area under the curve
BMI: body mass index
Cls: confidence intervals
DD: lumbar degenerative disease with diagnostic doubt
ECO: effective clinical outcome
JOA: Japanese orthopaedic association
LDD: lumbar degenerative disease
NECO: non- effective clinical outcome
NLR: negative likelihood ratio
ODI: Oswestry disability index
ORs: odds ratios
PLR: positive likelihood ratio
SD: standard deviation
SLR: single leg raise
SNRB: selective nerve root block
VAS: visual analog pain scale
